# Bio- and chemocatalysis cascades as a bridge between biology and chemistry for green polymer synthesis

**DOI:** 10.1186/s11658-017-0061-1

**Published:** 2017-12-04

**Authors:** Aleksandra Marszałek-Harych, Dawid Jędrzkiewicz, Jolanta Ejfler

**Affiliations:** Faculty of Chemistry, University of Wrocław, 14 F. Joliot-Curie, 50-383 Wrocław, Poland

**Keywords:** Polylactide, Lactic acid, Ring opening polymerization

## Abstract

The development and integration of bio- and chemocatalytic processes to convert renewable or biomass feedstocks into polymers is a vibrant field of research with enormous potential for environmental protection and the mitigation of global warming. Here, we review the biotechnological and chemical synthetic strategies for producing platform monomers from bio-based sources and transforming them into eco-polymers. We also discuss their advanced bio-application using the example of polylactide (PLA), the most valuable green polymer on the market.

**This article was specially invited by the editors and represents work by leading researchers.**

## Introduction

Concerns about the effects of the chemical industry on global warming and fossil fuel shortages have led to a considerable increase in consumer demand for sustainable, “green” chemicals. A focus on developing commercially viable processes that convert bio-derived feedstocks into chemicals is one response to this [1–3]. Because the vast majority of commonly used polymers are derived from petrochemical resources and not readily degradable or recyclable, the polymer industry in particular is actively exploring and integrating new technologies. Biodegradable and/or renewable polymers are seen as one long-term solution for the environmentally damaging impact of petro-polymer and plastic pollution [4–6].

Significant progress has been made in the development and implementation of new processes and technologies to convert renewable feedstock into new green polymers. The aliphatic polyesters are a prominent group. They include polylactides (PLAs), which is the major synthetic green polymer on the market [7, 8].

PLAs are highly biocompatible due to their ability to be bioresorbed via the Krebs cycle and show benign performance in life-cycle assessments (LCA). PLAs have been used in the production of environmentally friendly packaging, disposable products, and advanced materials with biomedical applications. Their current industrial production level is 180·10^3^ t with a forecast for 2020 of 1·10^6^ t. This forecast might even be too conservative, considering that PLAs could replace PET in 20% of its current applications [9, 10].

Unfortunately, large-scale industrial production of PLAs via ring-opening polymerization (ROP) of lactide is inefficient, with high feedstock requirements and manufacturing costs [11, 12]. Current lactide monomer production is very costly. A novel synthetic strategy for lactide formation from lactic acid would need to be cheap and simple to positively impact large-scale production of PLA.

Since the synthesis of lactide monomers begins with anaerobic fermentation of renewable sugars to lactic acid, a new, cheaper production method using emerging chemical catalytic routes and/or concurrent biotechnological processes appears promising.

Unlike lactide, the five-membered γ-butyrolactone (γ-BL) is a biomass-derived cyclic ester that would be a valuable monomer for the synthesis of the newly developed biopolyester poly(γ-butyrolactone) (PγBL), which has unique properties desirable for synthetic biomaterials. Although γ-BL is accessible from biomass sources, its polymerization has proven difficult [13]. A cheap biotechnological process via lipase-catalyzed ROP of γ-BL yielded only a mixture of oligomers. Chemically, γ-BL has long been referred to as a non-polymerizable monomer because of its low strain energy [14, 15]. It should also be noted that γ-BL is a List I Chemical under the Controlled Substances Act (CSA).

PγBL has recently been synthesized as both a linear and a cyclic topology using ROP in the presence of metal complexes as initiators, but this is thus far only possible on a laboratory scale [16].

Another desirable feature of PγBL is the complete thermal recyclability from the polymer back to its cyclic monomer without the formation of its hydrolysis product, γ-hydroxybutyric acid, which is an FDA-regulated substance. This makes it the only example of a completely recyclable biopolymer. Thermal or catalytic degradation of PLAs produces many kinds of products, such as lactic acid and linear and cyclic oligomers, but never only the lactide monomer.

Biopolymers can also be produced directly from sugars or lipids via bacterial fermentation. The most recognized examples are polyhydroxyalkanoates (PHAs). There is increasing interest worldwide to scale up the microbial production of PHAs. One of the most explored examples is the fermentation-based production of copolyesters from 3-hydroxybutyrate and 3-hydroxyvaleric acid [17–26] A chemical catalytic process would be more effective, but efficient catalysts such as those used for ROP of lactides are unavailable, although exciting success has been achieved in laboratory-scale synthesis [27–31].

While the US Department of Energy ranks lactic acid and γ-BL among the top 12 biomass-derived compounds best suited to replace petroleum-derived chemicals, bio-based chemical production is often limited by a lack of efficient conversion technology, especially when compared to the efficiency of processes conducted in the petrochemical industry [32–39]. Considerable attention is given to the design and development of such bio-based technologies.

Our intention here is to present a perspective on the preparation of green polyesters using integrated bio- and chemocatalytic processes. We will summarize the state of knowledge on monomer synthesis and controlled polymerization and look at unique applications. In terms of processes, we will also present potential methodological bridges that could connect biology and chemistry for efficient and sustainable polymer synthesis.

### Synthesis of renewable monomers

There are three major strategies to produce monomers or their precursors from natural resources. The most popular synthetic pathway is direct transformation of biomass into monomers via fermentation of carbohydrates. Another involves chemical degradation and transformation of natural polymers, exemplified by lignocellulosic biomass, which is the most abundant renewable polymer and which represents a sustainable feedstock. The last is based on a chemical transformation of organic compounds obtained directly from nature, such as vegetable oils, terpenes and resin acids.

### Synthesis of lactide precursor

**Scheme 1:**
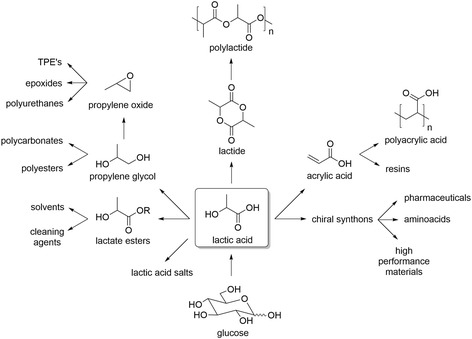
Chemical portfolio of lactic acid

Lactic acid is the most recognized bio-based chemicals. It is one of the most relevant platform molecules for the synthesis of multiple commodities and intermediate downstream chemicals (e.g., acrylic acid, 1,2-propanediol, pyruvic acid, acetaldehyde, 2,3-pentanedione, lactate ester and propylene glycol) that are already part of the “traditional chemistry portfolio”. Importantly, lactic acid can also be polymerized into biodegradable plastics like PLA (Scheme **1**) [40–46].

#### Fermentation of carbohydrates

The dominant commercial production method for lactic acid is anaerobic batch fermentation of glucose or sucrose using organisms such as *Lactobacillus delbrueckii*. Other microorganisms and biomass sources are also possible [42, 44]. Bacterial fermentation is the preferred industrial process used by NatureWorks LLC and Corbion, the two major producers of PLA. The classic process requires strict temperature (< 313 K) and pH (5–7) and involves isolation of the product followed by purification through subsequent esterification, distillation and hydrolysis. The problem in this industrial bioprocess is the disposal of waste generated by the neutralization of the calcium lactate intermediate.

Alternative technologies based on desalting and electrodialysis and/or using engineered yeast species such as *Pichia stipites* to ferment xylose have been examined [42, 45]. This process offers the possibility to convert lignocellulosic sugars and perform the fermentation at lower pH. Commercial use of lignocellulosic biomass to produce lactic acid demands microorganisms that can use all the sugars derived from lignocellulosic biomass.

In general, advancing the biotechnology needed for lactic acid production requires: (i) optimization of the bioconversion of carbohydrates, (ii) bioprocesses with high rate and yield, (iii) pH and inhibitor tolerance, and (iv) engineering of organisms to produce high-quality product from the biomass sources in a single bioreactor. Moreover, the low productivity of such processes may be a great obstacle to the expansion of this technology to fulfill the future demand for lactic acid.

#### Cascade chemocatalysis

Non-fermentative catalytic chemical transformations for lactic acid production are simpler and more efficient than the biotechnological processes. Several studies have focused on the conversion of biomass resources, such as glucose, fructose, cellulose, and trioses like dihydroxyacetone (DHA), to lactic acid through catalyzed routes [47–59] However, the yield of the lactic acid is far from satisfactory while using cellulose feedstock. The alternative chemical synthesis strategies are: (i) hydrothermal conversion of glycerol mediated by base catalysts [47, 53, 54]; (ii) conversion of cellulose feedstock or dihydroxyacetone catalyzed by Lewis acids [49, 55, 56]; (iii) conversion of glycerol by metal salts or metal complexes [52, 57, 58]; and (iv) conversion of glycerol in the presence of nanoparticle catalytic systems [59].

The alternative to biocatalytic synthesis of lactic acid is the novel cascade process containing bio- and chemocatalytic steps. It uses glycerol from biodiesel production as a raw material [51].

A synthetic strategy based on Lewis acid-catalyzed isomerization of 1,3-dihydroxyacetone (DHA) has been explored [47–53]. Lanthanum, lead salts and iridium complexes have been used as active catalysts in the reaction [48, 52]. However, issues with the separation of lactic acid from the reaction mixture and the toxicity of the catalysts led to solid catalysts such as zeolites gaining more interest. The new hybrid bio−/chemocatalytic synthetic pathway is based on the enzymatic oxidation of glycerol to DHA followed by isomerization to lactic acid in water.

**Fig. 1:**
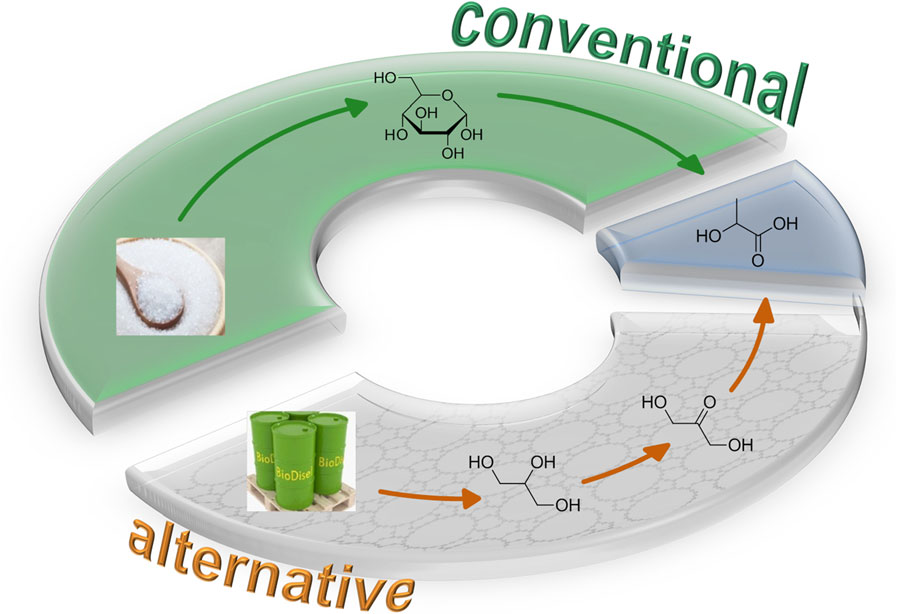
Conventional and alternative lactid acid synthesis

This process is particularly efficient in the presence of tin-containing MF1 zeolites, which are selective, recyclable and can be prepared by scalable methods (Fig. 1). Zeolite catalysts can also operate in concentrated aqueous or alcohol solutions, and it is possible to obtain two products, such as lactic acid or alkyl lactates. Additionally, the process contains the enzymatic production of dihydroxyacetone derived from crude glycerol, which is important from the point of view of the LCA.

Overall, cascade bio- and chemocatalytic production of lactic acid from glycerol seems ecologically and economically much more attractive than traditional biotechnological processes. It also appears to have a strong industrial implementation potential.

#### Recycling of polyactide

The recycling of polylactide to lactic acid via hydrolytic degradation has been also extensively studied [60–71]. However, the most success has been achieved in catalytic alcoholysis of PLA waste to alkyl lactates. Although the concept of alkyl lactate synthesis from PLA is well established on a laboratory scale, the application in industry is limited.

There was a recent report of a new simple method of PLA recycling to alkyl lactates using alcoholysis under solvothermal conditions. The best catalytic activities were obtained in the presence of magnesium and calcium alkoxides, which acted as catalysts synthesized in situ from organometallic or metallic precursors and an alcohol [68].

### Lactide synthesis

The conventional industrial process for converting lactic acid to lactide has two stages: (i) polycondensation of lactic acid to oligolactides, (ii) endothermic transesterification, which is facilitated in the presence of non-recoverable metal salts [69–71].

The removal of lactide by distillation is necessary to avoid the thermodynamically favored reverse reaction. An additional complication in that two-step process is the inconvenient purification method. An ideal one-step synthesis should involve lactic acid dimerization to lactyl lactate followed by its direct cyclization to one exclusive desired product lactide.

**Fig. 2:**
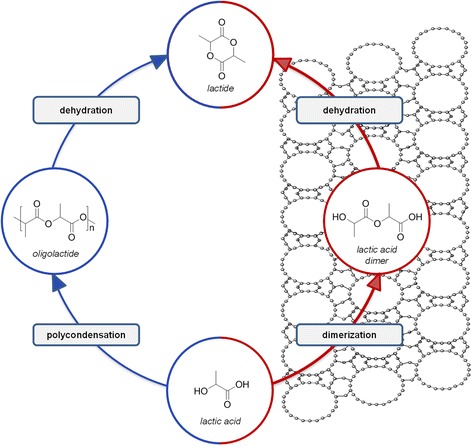
Lactide synthesis by classical and new routes in the presence of zeolite catalysts

A recent report focused on the novel direct zeolite-based catalytic process conversion of bio-based lactic acid to lactide (Fig. 2) [70]. The rationale for the use of zeolite catalysts is their ability to facilitate condensation reactions and the ideal recognition between molecular size and functionality differences between oligolactides and cyclic ester–lactides.

This method’s synthesis of lactide is fast and directly selective with the use of microporous 12-membered ring H-zeolites under continuous water removal. The shape-selective properties of zeolites are essential to a high yield of lactide, and the method outperforms the classic multistep process and avoids both racemization and side-product formation. This versatile zeolite technology may facilitate the synthesis of a wide range of renewable, high-performing, degradable bio-based polymers.

### Polymer synthesis

PLA is a versatile compostable polymer made from 100% renewable resources. The life cycle of PLA starts with renewable resources: a starch- or sugar-rich feedstock from the cheapest locally planted crops. Dextrose is converted to lactic acid via fermentation and a series of purification steps [11, 12]. The new and improved biotechnology proposes the conversion of cellulose or hemicellulose into fermentation sugars in a so-called biorefinery [12]. Lactic acid is transformed into lactide, which is then polymerized and processed into PLA pellets.

**Fig. 3:**
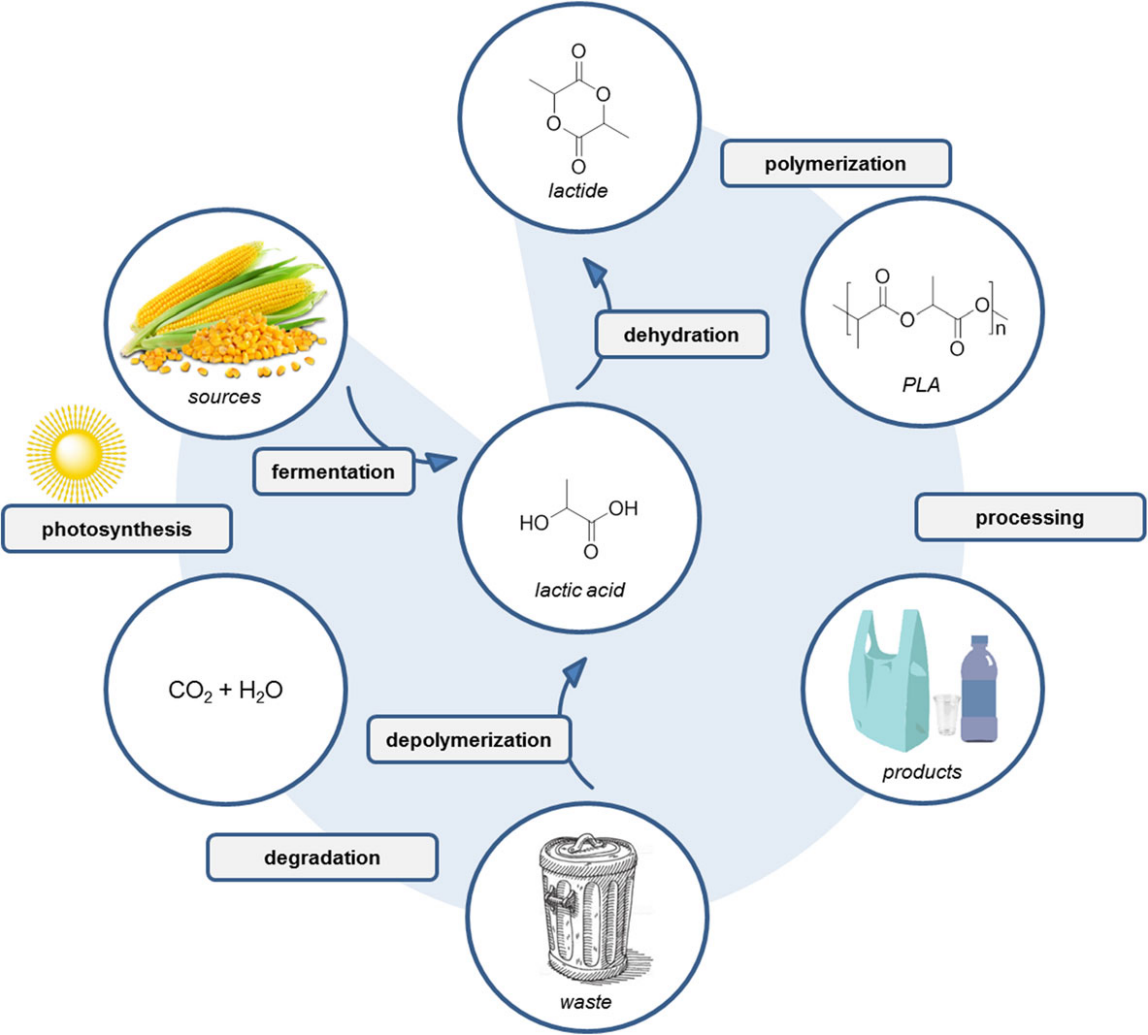
The LCA of PLA

Optimally, the LCA should include all input and output aggregated in a series of categories extending from the production of raw materials to the final disposal of possible consumer products. A schematic of the LCA of PLA is shown in Fig. 3.

ROP of bio-derived cyclic esters such as lactide is the best synthetic strategy to prepare aliphatic biopolyesters with desired and strictly planned properties (Fig. 1) [72–76]. This is a widely used method to prepare well-defined biopolyesters with competitive applications. Some of the essential parameters to ensure living ROP are polymer microstructure, predictable chain length, dispersity (PDI) and planned chain ends. The control of those parameters is essential for the design of the physical, mechanical and degradation properties of the PLA.

Enzymatic ROP of lactide is possible but its success is limited in comparison to other chemical catalytic species. Thus far, the most advanced are the so-called single-site initiators based on metal complexes that reach very high levels of activity and stereocontrol of ROP processes [72–76].

**Fig. 4:**
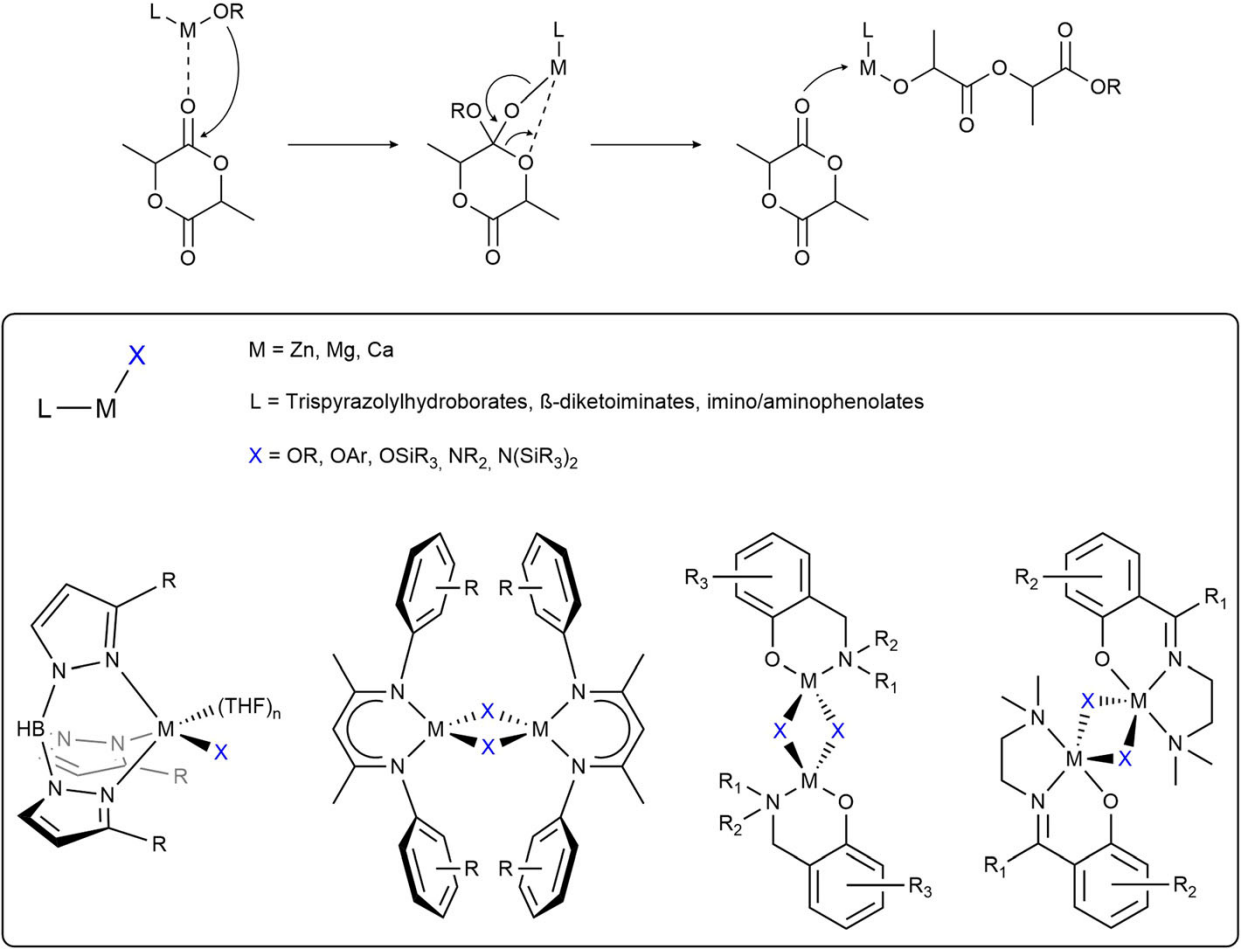
ROP of lactide (top), single-site initiators used in ROP of cyclic esters (bottom)

The general formula of active in ROP metal complexes is L-M-OR, where L is the ancillary ligand(s), M is metal center, and OR is the initiating group (Fig. 4). The aminophenol auxiliaries are currently the most popular [77–98]. In the context of bio-applications, complexes including benign metals (Zn, Mg, Ca) are still the most desired [77–98]. However, for those metals, the synthesis of heteroleptic complexes with a single-site motif is very difficult because they easily undergo ligand redistribution reactions [81, 84]. We recently proved that the precise synthesis of the desired L-M-OR/(L-M-OR)_2_ complexes with kinetically labile metals (Ca, Mg, Zn) relates to the molecular fitting of both ancillary (L) and initiating (OR) ligands. That is the guideline for rational structural motif design of new initiators for ROP reactions [77–79, 94–97].

Another alternative is the binary catalytic system with the L_2_M/ROH combination. Some of these have a tendency to indicate selectivity towards polymerization or alcoholysis of lactides, which could lead to synthesis of alkyl esters or oligomers [97].

### Polymer therapeutics

Polymer therapeutics encompass supramolecular drug-delivery systems and drug/protein–polymer conjugates. Although several polymer–protein and polymer–low molecular drug conjugates have received market approval, the search for new drug-delivery concepts and new courses of action is the major driving force in polymer therapeutics [99–101].

Bio-applicable polymers should have advanced properties and functions but also comply with regulations on biocompatibility. There is a huge gap between the promising biopolymers reported in the literature and fully developed bio-applicable systems. The overall number of approved polymers matrices for human use is relatively low. Biodegradable aliphatic polyesters, like PLA, polyglycolide (PGA) and poly(lactide-co-glycolide) (PLGA), are widely used in life sciences in a variety of bio-applications, including controlled drug release, gene therapy, regenerative medicine and implants [102, 103].

The copolymer PLGA is the most widely used material for drug release systems. Major problems encountered in controlled drug-delivery systems based on biodegradable PLGA matrices are the overall bioavailability of the released drugs and the rapid initial release from the polymer carriers. Therefore, there is still great interest in a new, effective and safe delivery system for the delivery of labile and/or large drug molecules to specific targets.

**Fig. 5:**
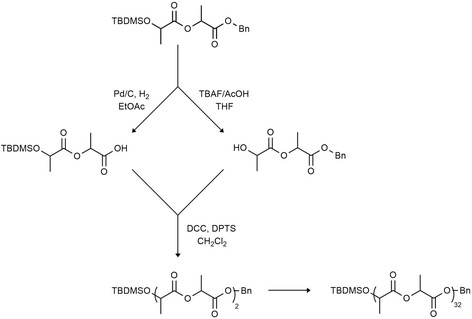
The iterative method for oligolactide synthesis

The controlled synthesis of PLA, PGA and PLGA is accomplished through metal-catalyzed/−initiated ROP of cyclic esters like lactide and glycolide [72–98, 104, 105]. The molecular microstructure of synthetic polymers and copolymers is not as sophisticated and precisely designed as those typical for biopolymers. The most precise existing methodology for PLGA synthesis relies on the stepwise coupling of monomer units via an iterative method: an example of oligolactide synthesis is presented in Fig. 5 [106].

This approach is valuable but has limitations. Each coupling step should be nearly quantitative, with protecting groups required to control the reactivity of the monomers; and time-consuming cycles involving coupling, washing and deprotection steps are needed for each monomer attachment.

Alternatively, catalytic living polymerization of precisely designed monomers could enable the synthesis of well-defined and complex macromolecular architectures. Nowadays, controlled living polymerizations, such as RAFT, ATRP, NMP and ROP, produce homopolymers with defined molecular weights and end groups, but incorporation of the new co-monomer in a fixed region of the polymer chain is difficult.

**Fig. 6:**
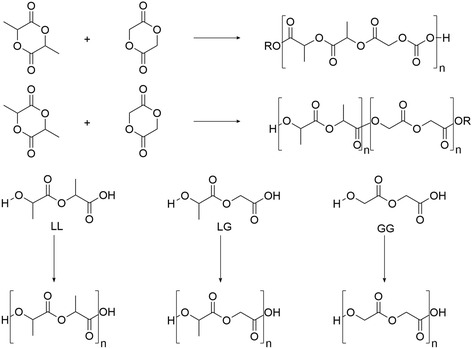
(Top) Ring-opening polymerization (ROP) used to prepare repeating sequence (ideal) or random (real) copolymer. (Bottom) Segmer-assembly polymerization (SAP) used to prepare sequence polymers. L – lactic unit, G – glycolic unit

A segmer-assembly polymerization (SAP) approach was recently proposed. This procedure leads to the formation of periodic copolymers and allows access to a variety of PLGA sequences (Fig. 6) [107]. However, this method is less efficient than ROP.

**Fig. 7:**
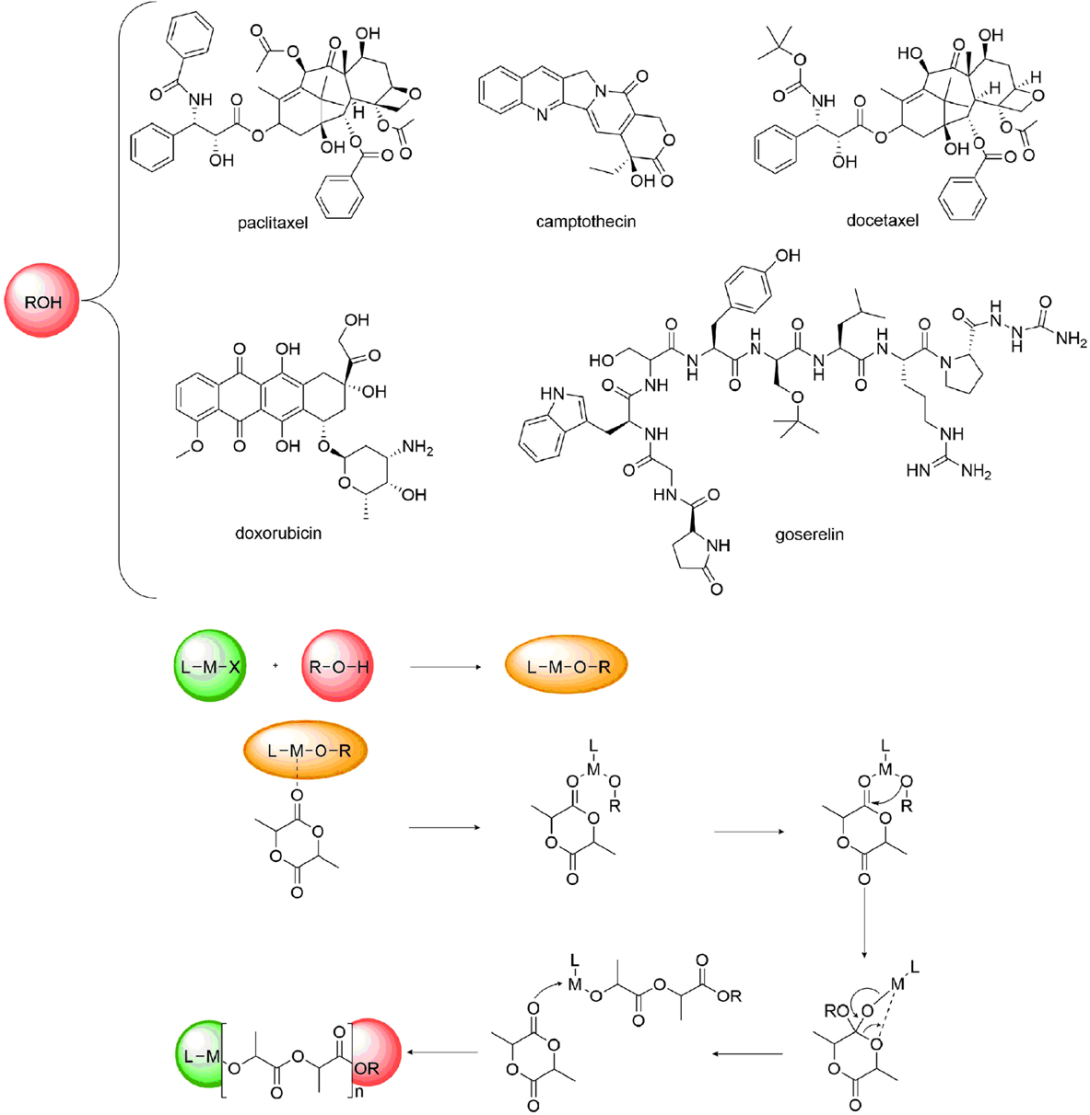
Drug-initiated methodology for the synthesis of polymer-drug conjugates

A new concept based on ROP of cyclic esters is the design of simple drug delivery systems, obtained by the drug-initiated method, which allows for synthesis of well-defined pro-drugs. The method consists of growing short polymer chains and drugs bonded to the polymer as an end group [108–110]. The resulting materials obtained in a well-controlled ROP process contain all polymer chains with a similar molar mass and the same end group, which is one drug molecule. The simple synthetic strategy means a potentially easy scale-up, which would be a crucial advantage compared to conventional drug-delivery systems. The strategy applies to hydroxyl-containing drugs, which are used as initiating groups during ROP. The examples are well-established anticancer drugs, such as paclitaxel, docetaxel, camptothecin, doxorubicin and goserelin, and immunosuppressive agents, such as cyclosporine A (Fig. 7).

These polymer–drug conjugates can be obtained in a one-pot synthetic strategy and easily self-assembled into polymer nanoparticles, incorporated into lipid nanocarriers, or be applicable as polymer drugs. What’s more, these systems could be obtained by modular construction of polymer chains, with the copolymer backbone indicating new properties or functions.

## Conclusions

Synthetic polymers are still chosen based on their physicochemical and structural properties. Controlled polymerization provides a promising platform to produce high-performance polymers with controlled molecular weights, functionalities and molecular architecture. Sustainable biopolymer production is complicated, with the main challenges being:

- Monomer production from biomass sources
- Accessibility of economic and ecological polymerization processes
- Availability of suitable bio- and chemocatalysts for ROP
- Availability of recycling technology to regenerate polymer waste to its cyclic monomer

In the nearest future, the bio- and chemocatalysis cascade will constitute the cornerstone of any strategy to realize the objectives for green technologies. Although many bio- and chemocatalytic systems can catalyze different transformations, performance improvements and their integration and transfer to larger-scale processes are still needed.

Ultimately, creating a sustainable renewable polymer industry should involve cooperative efforts between the chemicals industry, biomass conversion companies, and academic research groups. The aim should be to identify valuable novel conversion processes that can use the existing infrastructure to upgrade biomass monomers.

With continuous efforts to develop new biological and chemical technologies, and improved acceptance and understanding from the public, renewable polymers will play an increasing role in sustainable production and in the promotion of more environmentally benign materials.

## Abbreviations

ATRP: Atom-transfer radical-polymerization
DHA: Dihydroxyacetone
FDA: Food & Drug Administration
LCA: Life-cycle assessment
NMP: Nitroxide-mediated radical polymerization
PDI: Polydispersion index
PET: Polyethylene terephthalate
PGA: Polyglycolide
PHA: Polyhydroxyalkanoate
PLA: Polylactide
PLGA: Poly (lactide-co-glycolide)
PγBL: Poly (γ-butyrolactone)
RAFT: Reversible addition-fragmentation chain-transfer polymerization
ROP: Ring-opening polymerization
SAP: Segmer-assembly polymerization
γ-BL: γ-butyrolactone
